# Fecal microbiota transplantation improves functional constipation through the gut microbiome–bile acid–receptor axis

**DOI:** 10.3389/fmed.2026.1751593

**Published:** 2026-02-09

**Authors:** Dongxu Wen, Shuzhen Liu, Yukun Wu, Haixuan Zhang, Kun Zhang

**Affiliations:** 1 Shandong University of Traditional Chinese Medicine, Jinan, China; 2 First Teaching Hospital of Tianjin University of Traditional Chinese Medicine, Tianjin, China; 3 Beijing International Studies University, Beijing, China; 4 Guangzhou University of Chinese Medicine, Guangzhou, China; 5 Linyi Hospital of Traditional Chinese Medicine, Linyi, China

**Keywords:** bile acids, Farnesoid X receptor, fecal microbiota transplantation, functional constipation, Takeda G protein–coupled receptor 5

## Abstract

Functional Constipation (FC) is a prevalent gastrointestinal motility disorder worldwide that markedly impairs patients’ quality of life, yet the currently available treatment options often show limited efficacy. In recent years, research has gradually revealed the critical role of the gut microbiota and bile acid metabolism in the pathogenesis of FC. Fecal Microbiota Transplantation (FMT), which restores the intestinal microecological balance by transferring gut microbiota from healthy donors, has demonstrated clinical efficacy in promoting bowel movements, improving stool consistency, and enhancing patients’ quality of life. However, its underlying mechanisms remain incompletely understood. Current evidence indicates that FMT restores microbial diversity, increases beneficial taxa, and partially reconstructs the bile acids (BAs) profile, thereby modulating Farnesoid X Receptor (FXR) and Takeda G Protein–Coupled Receptor 5 (TGR5) mediated signaling pathways to enhance intestinal secretion and alleviate constipation-related symptoms. The resulting microbiota–bile acid–receptor pathway elucidates the mechanistic link between microbial remodeling and host gastrointestinal motility, thereby offering theoretical support for the therapeutic application of FMT in functional constipation.

## 1 Introduction

Functional Constipation (FC) represents one of the most common subtypes of constipation in clinical practice, is typically defined by decreased bowel movement frequency, prolonged or difficulty in evacuation, and a sensation of incomplete defecation. The global prevalence of this condition is approximately 12% (1). Beyond affecting daily life, FC has been strongly associated with metabolic derangements as well as psychological comorbidities, including anxiety and depression (2, 3). Current therapies such as laxatives and prokinetics often provide suboptimal and transient relief, underscoring the need for alternative strategies (4). It is thus of considerable clinical significance to explore new therapeutic modalities.

Accumulating evidence indicates that alterations in gut microbiota structure and function play an essential role in the onset and progression of FC. Patients with FC often exhibit reduced microbial diversity, accompanied by a deficiency of functional bacteria responsible for producing short-chain fatty acids (SCFAs) and secondary bile acids (BAs) (5). Bile acid, especially secondary bile acid, was demonstrated to modulate the intestinal motor function and mucosal function primarily through the activation of two key bile acid receptors: Farnesoid X receptor (FXR) and Takeda G protein–coupled receptor 5 (TGR5) (6). Fecal Microbiota Transplantation (FMT) has recently been recognized as a promising therapeutic approach for Functional Constipation (FC) and related disorders, demonstrating improvements in bowel function and enhancements in quality of life (7). However, despite encouraging clinical outcomes, the mechanisms by which FMT exerts its benefits remain incompletely understood. This review synthesizes current evidence on how FMT influences the gut microbiota–BA–receptor axis and its relevance to FC pathophysiology, and then correlate these influences and clinical symptom improvements, so as to lay a basis of FMT application at the level of theory.

## 2 Changes in intestinal microbiota after FMT intervention

### 2.1 Changes in microbiota diversity due to FMT

FMT restores intestinal microecological balance by introducing donor microbiota into the recipient’s gut, thereby significantly improving the intestinal microbial environment. This includes enhancing *α*-diversity (richness and evenness of microbial communities) as well as *β*-diversity (differences in microbial composition among populations). Specifically speaking, FMT expands *α*-diversity of the receiver’s gut, and the microbiota profile of the receiver becomes more similar to healthy donor microbiotas. Increased *α*-diversity is positively correlated with increased bowel movement frequencies among FC patients. Furthermore, the restoration of α-diversity is often accompanied by an increase in key metabolic products, such as short-chain fatty acids (SCFAs), which are instrumental in sustaining intestinal barrier homeostasis and improving bowel function (8, 9). And *β*-diversity FMT expands similarity among donor and receiver microbiotas. It’s manifested not only at the general level of the structure of the community, but at transplantation and steady establishment of key microbial species, respectively (10, 11). After FMT, for example, there’s reappearance of *Faecalibacterium prausnitzii* in the receiver’s gut, and it’s closely connected with clinical symptom amelioration (7). Donor source of FMT and treatment times exert significant influences on the scale of restoration. Healthy donor sources tend toward more lasting restoration of improvements, and repeat transplantation achieves superior steady reconstruction of microbiotas toward a single transplantation (7, 12).

### 2.2 Changes in microbiota structure

In patients with FC, the gut microbiota imbalance is not only reflected in the overall reduction of diversity but also in specific structural changes. Studies have shown that common characteristics of this imbalance include an increased Firmicutes/Bacteroidetes ratio, reflecting increased Firmicutes abundance, decreased Bacteroidetes, along with an increase in certain opportunistic pathogens, while beneficial bacteria are reduced (13, 14). At the family and genus levels, microbiota associated with short-chain fatty acid (SCFA) production, such as Muribaculaceae and Bacteroides, tend to decrease, while certain opportunistic pathogens, including Enterobacteriaceae and Clostridium sensu stricto, tend to increase. These changes are closely related to gastrointestinal motility disorders (15, 16).

Following FMT intervention, these structural imbalances gradually recover. At the phylum level, the ratio of Firmicutes to Bacteroidetes tends to normalize, reflecting the reconstruction of intestinal homeostasis. Notably, at the family level, increases in the abundance of key taxa such as Prevotellaceae and Ruminococcaceae, contribute to enhanced the production of short-chain fatty acids (SCFAs) and secondary bile acids. At the genus level, there is a marked restoration of Faecalibacterium and Roseburia, both of which are butyrate-producing bacteria. Butyrate serves as the main energy substrate for colonic epithelial cells, promotes intestinal motility, and enhances barrier function (17). Meanwhile, the proportion of opportunistic pathogens decreases (16, 18). These changes suggest that FMT reshapes the microbiota composition, restores metabolic function, and establishes a foundation for balancing the downstream bile acid profile and activating receptor signaling. Table 1 summarizes the major effects of FMT on gut microbiota in patients with FC (Table 1).

**Table 1:** Major effects of fecal microbiota transplantation on gut microbiota diversity and composition.

Microbiota level	Changes in FC patients	Post-FMT changes	Potential mechanisms
α-diversity	Reduced richness and evenness	Increased, closer to donors	Restoration of ecological stability
β-diversity	Distinct from healthy controls	Shifted toward donor profile	Reconstruction of overall community balance
F/B ratio	Elevated (Firmicutes↑, Bacteroidetes↓)	Normalized	Modulation of SCFA production and motility
Butyrate producers (e.g., Faecalibacterium, Roseburia)	Decreased	Increased	Enhanced butyrate supply; improved epithelial energy and barrier
Conditional pathogens (e.g., Enterobacteriaceae, Clostridium sensu stricto)	Increased	Decreased	Reduced pro-inflammatory activity and harmful metabolites
Other key taxa (e.g., Bacteroides, Prevotella)	Bacteroides↓, Prevotella dysregulated	Partially restored	Re-establishment of bile acid metabolism and polysaccharide degradation

FC, functional constipation; FMT, fecal microbiota transplantation; F/B, Firmicutes/Bacteroidetes; SCFAs, short-chain fatty acids.

In addition to functional constipation, evidence from other clinical contexts suggests that fecal microbiota transplantation can induce comparable shifts in gut microbial composition. In the study by DuPont et al., which examined microbiota changes following FMT in PD patients, the genera Roseburia and *Ruthenibacterium* became among the 10 most prevalent taxa during and after treatment, although they were not among the dominant genera at baseline. Roseburia belongs to the butyrate-producing Lachnospiraceae family (Firmicutes phylum), while *Ruthenibacterium* spp. are members of the Ruminococcaceae family, well known for producing short-chain fatty acids and contributing to gut barrier integrity and immune function. *Ruthenibacterium* spp. have previously been detected in the intestinal microbiota of PD patients. In addition, Collinsella emerged as one of the most common genera following FMT. Collinsella (Actinomycetota/Actinobacteria) has been associated with protection against SARS-CoV-2 infection and was shown to decrease during weight-loss interventions in obese patients with type 2 diabetes. Other Firmicutes taxa, including *Limnochordaceae*, *Peptostreptococcaceae*, and *Lactobacillaceae*, also increased significantly after FMT. However, there is currently no evidence indicating that increased abundance of members of the *Limnochordaceae* family confers specific health benefits (19).

## 3 Changes in bile acid profile

### 3.1 The impact of FMT on the balance between primary and secondary bile acids

Bile acid metabolism relies on the critical process of the enterohepatic circulation, in which most bile acids are reabsorbed in the terminal ileum via the portal circulation and transported back to the liver (20). Only a small proportion of primary bile acids, such as Cholic Acid (CA) and Chenodeoxycholic Acid (CDCA), escape absorption in the small intestine and enter the colon. There, through enzymatic reactions such as 7α-dehydroxylation mediated by bacterial genera including *Clostridium scindens*, they are converted into secondary bile acids, including Deoxycholic Acid (DCA) and Lithocholic Acid (LCA) (21, 22). In healthy individuals, secondary bile acids typically dominate the fecal bile acid profile. This is dependent on the cooperative actions of various microbiota species, such as those carrying bile salt hydrolase (BSH), which catalyze the deconjugation reaction and provide substrates for subsequent 7α-dehydroxylation (23, 24).

Research evidence indicates that patients with constipation often exhibit an increased proportion of primary bile acids and a marked reduction in secondary bile acids, suggesting a functional impairment in the bile acid conversion process (25). This imbalance not only indicates the loss of key functional microbiota but may also lead to insufficient activation of downstream receptors such as FXR and TGR5, then affecting intestinal motility and secretory function. FMT can partially restore this imbalance. Experimental studies have shown that FMT elevates secondary bile acid concentrations while reducing primary bile acid proportions in recipient feces, resulting in a bile acid profile that gradually approximates that of healthy donors (26–28). This suggests that FMT not only alters the microbiota composition but also restores the balance between primary and secondary bile acids by rebuilding microbial functional groups, thereby providing a foundation for the activation of FXR/TGR5 signaling and improvement in clinical symptoms.

In addition to primary and secondary bile acids, FMT may also influence other derivatives, such as ursodeoxycholic acid (UDCA). These metabolites play a potential role in maintaining gut barrier integrity and microbiota homeostasis. However, direct evidence linking these metabolites to constipation remains limited (29). Table 2 summarizes the main primary and secondary bile acids, the microbial processes involved, and their major receptor pathways (Table 2).

**Table 2:** Primary and secondary bile acids, related microbiota, and major receptors.

Bile acid type	Representative molecules	Key taxa/enzymes	Major receptors	Physiological significance
Primary BAs	CA, CDCA	Synthesized in the liver; secreted as conjugates	FXR (mainly in ileal epithelium), TGR5 (partly)	Feedback regulation of BAs synthesis; maintenance of mucosal barrier
Secondary BAs	DCA, LCA	*Clostridium scindens* and other 7α-dehydroxylating bacteria; require cooperation of BSH-positive strains	TGR5 (enteroendocrine L cells, neurons), FXR (partly)	Activation of GLP-1/5-HT signaling; stimulation of motility and secretion
Other derivatives	UDCA, ω-MCA	Produced by various microbial transformations	FXR, TGR5	Anti-inflammatory effects; modulation of microbiota; potential motility improvement

Bas, bile acids; CA, cholic acid; CDCA, chenodeoxycholic acid; DCA, deoxycholic acid; LCA, lithocholic acid; UDCA, ursodeoxycholic acid; ω-MCA, ω-muricholic acid; BSH, bile salt hydrolase; FXR, Farnesoid X receptor; TGR5, Takeda G protein–coupled receptor 5; GLP-1, Glucagon-like peptide-1; 5-HT, 5-hydroxytryptamine.

### 3.2 The relationship between bile acids and receptor activation

The reestablishment of the ratio between primary and secondary bile acids restores secondary bile acids as effective agonists of TGR5, while maintaining the sustained stimulation of FXR by primary bile acids (30, 31). This indicates that the restoration of the bile acid profile is not only a metabolic outcome but also represents the reestablishment of receptor signaling pathways. Therefore, the therapeutic effect of FMT on FC can be understood as a progressive process: initially, bile acid metabolism is reestablished through microbial modulation, primary and secondary bile acids act as signaling mediators to activate FXR and TGR5 pathways. The following section will elaborate on the specific manifestations of this receptor activation pattern.

## 4 Activation of FXR/TGR5 receptors

Bile acids, beyond their digestive roles, also function as pivotal signaling molecules in the regulation of intestinal physiology. Among their receptors, FXR and TGR5 are particularly critical, as they not only maintain bile acid homeostasis but also play a central role in regulating intestinal motility. FMT can indirectly influence these signaling pathways by altering the bile acid profile. Several animal studies have consistently shown that after FMT, secondary bile acids increase in the recipient’s feces, leading to activates FXR and TGR5 in both the ileum and colon (6).

### 4.1 FXR activation pattern and its effects

After FMT, gut microbiota remodeling significantly increases the concentration of CDCA in the ileum (32). CDCA, as an endogenous ligand for FXR, can directly activate FXR (33). Once activated, FXR plays a pivotal role in maintaining bile acid homeostasis by inhibiting the activity of cholesterol 7α-hydroxylase (CYP7A1), while simultaneously facilitating the generation of primary bile acids, including CA and CDCA (34). Furthermore, FXR activation in intestinal epithelial cells stimulates fibroblast growth factor 19 (FGF19) secretion, thereby establishing a “gut–liver axis” feedback loop. This axis further fine-tunes hepatic bile acid synthesis and integrates bile acid signaling with energy metabolism (35).

### 4.2 TGR5 activation pattern and its effects

Representative secondary bile acids, including DCA and LCA, exhibit high binding affinity for TGR5 and activate this receptor when they accumulate in the colon (36). Activation of TGR5 stimulates the synthesis of cyclic Adenosine Monophosphate (cAMP) through the Gs protein–coupled signaling pathway, which subsequently leading to activation of downstream Protein Kinase A (PKA) (37), ultimately inducing the expression and release of 5-Hydroxytryptamine (5-HT) (38).

5-HT, as a key neurotransmitter in the gut, not only promotes chloride and water secretion, softening stool (39), but also enhances smooth muscle contraction and colonic propulsive motility by stimulating the enteric nervous system. In addition, TGR5 activation stimulates enteroendocrine L cells to release Glucagon-like peptide-1 (GLP-1), which, similar to 5-HT, works in concert to promote motility and secretion (40). Clinical studies have further confirmed that, following FMT treatment, patients with constipation exhibit a significant increase in 5-HT levels within the colonic mucosa, along with enhanced 5-HT expression on intestinal epithelial cell surfaces, thereby effectively enhancing intestinal secretion and propulsive motility (41). Results from animal experiments demonstrate that in TGR5 knockout mice, the colonic propulsion rate increased by only 18% after FMT intervention, which was markedly lower than the 42% observed in wild-type mice (*p* < 0.05). This provides compelling evidence that TGR5 plays an indispensable role in the pro-motility effects induced by FMT (42, 43).

FXR and TGR5, both bile acid–dependent receptors, cooperate to maintain bile acid homeostasis and intestinal function. FXR activation can upregulate the expression of TGR5, and the coordinated activity of these receptors promotes the GLP-1 secretion from L cells, thereby further enhancing intestinal propulsive function (44). Additionally, the use of an FXR-selective agonist alone has limited effects on metabolic improvement, but when combined with TGR5 activation, it can more effectively correct bile acid imbalance and microbiota abnormalities (45).

### 4.3 Differences in receptor activation and subtype specificity

Although the involvement of FXR and TGR5 receptors has been confirmed in multiple studies, there are still discrepancies between studies. In animal experiments, both FXR and TGR5 are often activated after FMT (6). However, in clinical patients, the improvement in constipation symptoms following FMT is more strongly associated with the activation of the FXR pathway. This difference may be related to factors such as the donor microbiota composition, the patients’ bile acid levels, and the method of microbiota transplantation (46–48).

Additionally, the dysbiosis patterns of the microbiota differ across constipation subtypes, leading to variations in bile acid metabolites. In slow-transit constipation (STC), the deficiency of secondary bile acids results in impaired TGR5 signaling. FMT can restore its function by rebuilding microbiota that produce secondary bile acids (49, 50). On the other hand, outlet obstruction constipation (OOC) is often accompanied by localized inflammation and barrier damage, making it more reliant on compensatory FXR activation to maintain mucosal barrier integrity and bile acid homeostasis (51).

## 5 The link between the microbiota–bile acid–receptor axis and constipation improvement

FMT has been shown to significantly increase bowel movement frequency, with studies reporting that nearly 75% of patients achieve at least three spontaneous complete bowel movements per week after treatment and the Wexner Constipation Score (WCS) decreased from 12.12 ± 4.05 before treatment to 7.12 ± 3.52 after treatment (*p* < 0.05), indicating a significant reduction in the severity of constipation. Stool consistency also improved, with the Bristol Stool Form Scale transitioning from harder types (1–2) to softer types (3–4). In addition to improving bowel movements, FMT also enhanced the patients’ quality of life. The Gastrointestinal Quality of Life Index (GIQLI) score significantly increased, suggesting that patients experienced relief from symptom distress, psychological burden, and daily life impact (7, 48, 52).

**Figure 1:**
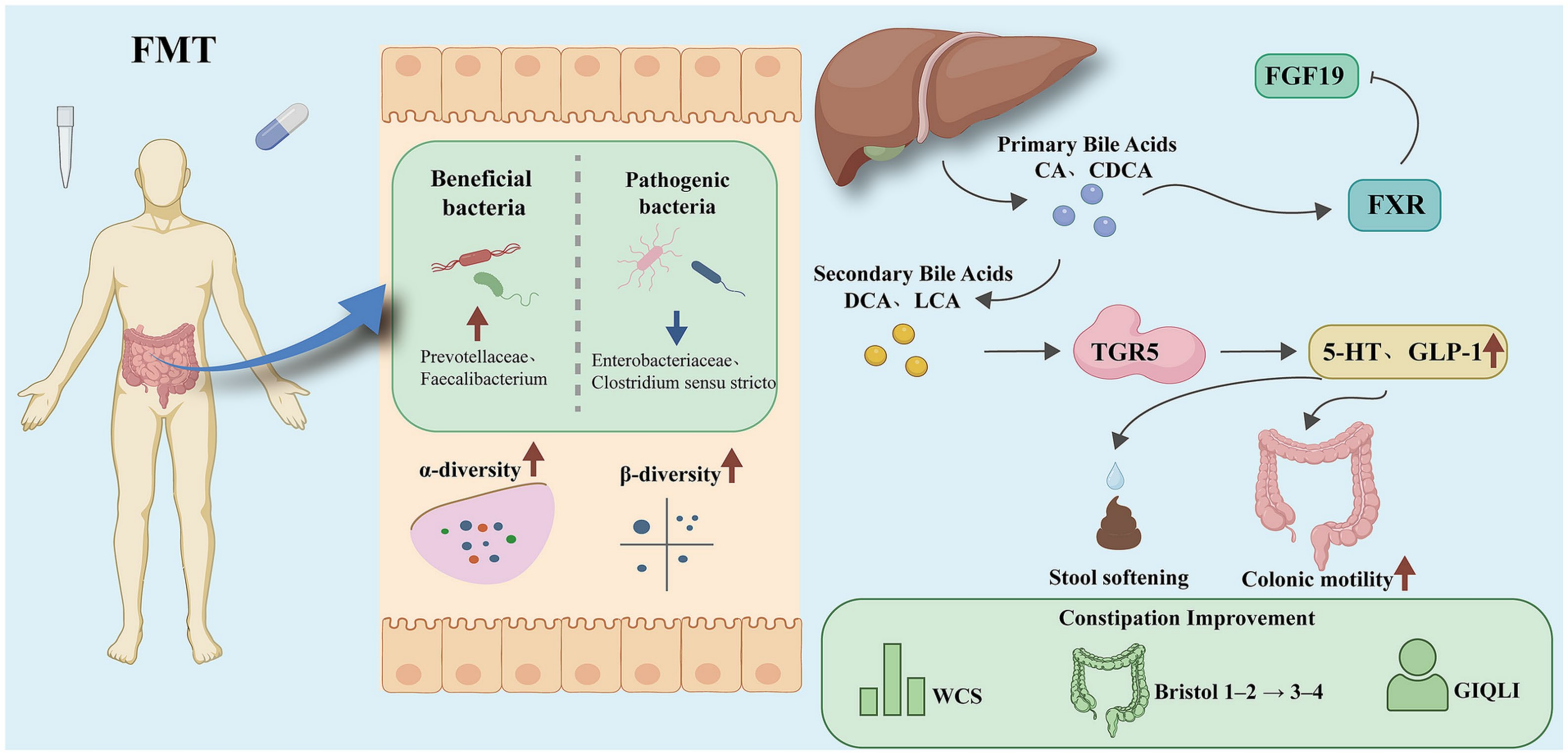
Schematic diagram of FMT improving FC through the “microbiota–bile acid–FXR/TGR5” axis.

At the mechanistic level, FMT reshapes the composition of the colonic microbiota (22), restores microbiota that produce secondary bile acids, and promotes the reactivation of the bile acid-mediated FXR/TGR5 signaling pathway. This process not only improves the distribution of microbiota-derived metabolites but also enhances intestinal barrier function and colonic propulsion, leading to a reduction in colonic transit time (CTT) (6, 26). While the specific microbiota–metabolite–receptor effect chain still requires further elucidation, existing evidence supports that FMT improves motility and alleviates symptoms through the “microbiota–bile acid–receptor axis.” It is noteworthy that although FMT demonstrates significant clinical efficacy in the short term, its long-term therapeutic effects remain somewhat unstable and uncertain. Follow-up studies have shown that some patients maintain symptom improvement for 3–6 months, while others gradually experience relapse (48). Therefore, the stability of the donor microbiota and its persistent colonization in the recipient’s gut are considered key factors determining long-term efficacy (53). Additionally, factors such as microbiota selection, diet, medications, and the host’s metabolic state may interfere with the colonization and maintenance of the transplanted microbiota. Optimizing donor selection, adopting multiple transplantation strategies, and assessing the baseline microbiota of the recipient could help improve the lasting efficacy of FMT (54, 55). The overall mechanism is summarized in Figure 1.

FMT alleviates constipation via the gut microbiota-bile acid-FXR/TGR5-5-HT axis. Overall mechanism framework of Fecal microbiota transplantation (FMT) in functional constipation. FMT reshapes gut microbiota composition by increasing beneficial taxa (e.g., Prevotellaceae, Faecalibacterium) and reducing pathogenic taxa (e.g., Enterobacteriaceae, Clostridium sensu stricto), thereby restoring both *α*-diversity and *β*-diversity. Microbial remodeling facilitates bile acid metabolism reconstruction, characterized by reduced primary bile acids [cholic acid (CA), chenodeoxycholic acid (CDCA)] and increased secondary bile acids [deoxycholic acid (DCA), lithocholic acid (LCA)]. These metabolites activate bile acid receptors in distinct regions of the intestine: in the ileum, Farnesoid X Receptor (FXR) induces fibroblast growth factor 19 (FGF19) to maintain bile acid homeostasis, whereas in the colon, Takeda G Protein–Coupled Receptor 5 (TGR5) promotes the secretion of 5-Hydroxytryptamine (5-HT) and Glucagon-like peptide-1 (GLP-1). Through these pathways, stool softening, enhanced colonic motility, and improved bowel habits are achieved, reflected by reduced Wexner constipation scores (WCS), normalized Bristol stool scale (from type 1–2 to type 3–4), and higher gastrointestinal quality of life index (GIQLI).

## 6 Discussion and outlook

This article reviews the mechanisms by which FMT influences functional constipation, emphasizing the critical role of the microbiota–bile acid–receptor axis in symptom improvement. Existing evidence shows that FMT can significantly improve the diversity of the gut microbiota, reshaping its structure to more closely resemble that of a healthy donor (8, 16, 18). This creates favorable conditions for bile acid conversion an SCFA production.

The restoration of bile acid metabolism represents a critical mechanism through which FMT alleviates constipation. Patients with FC commonly exhibit an imbalance in bile acid composition, with elevated primary bile acids and reduced secondary bile acids (25). Through FMT, the concentration of secondary bile acids, such as DCA and LCA, is increased, thereby promoting intestinal secretion and motility while engaging bile acid receptors, TGR5 in the colon and FXR in the ileum, to modulate gut function. Animal studies also confirm that both FXR and TGR5 pathways are activated after FMT (6).

At the pharmacological level, research targeting bile acid receptors offers new perspectives for treating FC. FXR agonists, such as obeticholic acid, can improve the intestinal barrier by regulating bile acid synthesis (56). TGR5 agonists, like INT-777, enhance the secretion of 5-HT and GLP-1, promoting intestinal motility (57). In animal experiments, the dual agonist INT-767, which simultaneously targets FXR and TGR5, has been shown to produce a more pronounced pro-motility effect (58, 59). However, single-agent treatments have limitations in efficacy (60). Therefore, the combined application of FMT and targeted drugs holds promise in not only improving microbiota imbalance but also enhancing receptor signaling, potentially leading to more sustained and stable therapeutic effects.

Beyond functional constipation, evidence from other disease settings offers valuable complementary insights into the clinical outcomes of fecal microbiota transplantation. In a systematic review evaluating the safety of fecal microbiota transplantation (FMT) for the treatment of Parkinson’s disease (PD), it was noted that stool frequency and quality of life improved significantly in PD patients treated with FMT compared with control groups (61, 62). Evidence regarding changes in Bristol Stool Scale (BSS) scores, however, remains limited, and findings on stool consistency are inconclusive. Differences in alpha diversity between the FMT and placebo groups were less pronounced than changes in beta diversity. Reductions in levodopa-equivalent daily dose (LEDD) and in Beck Anxiety Inventory (BAI) scores were observed in the FMT group at 12 months and 6 months, respectively, compared with placebo. In contrast, complete bowel movements remained more frequent in the placebo group than in the FMT group up to 12 months (63).

At the same time, clinical outcomes following fecal microbiota transplantation show considerable variability across studies, underscoring limitations in the current evidence base. Non-Motor Symptoms Scale (NMSS) scores increased in the FMT group compared with placebo at 6 months in the study by Scheperjans et al., whereas in the study by DuPont et al., subjective non-motor symptoms assessed using visual analog scales improved in the FMT group. In the study by Segal et al., the follow-up duration was approximately 24 weeks. Overall, additional research is needed to clarify long-term efficacy, durability of response, and safety (63).

In addition to functional and metabolic alterations, intestinal barrier impairment and low-grade inflammation may also contribute to constipation-related symptoms. Bellini et al. reported that patients with Parkinson’s disease exhibit disrupted epithelial integrity accompanied by gut microbiota dysbiosis and enteric glial activation. Although derived from Parkinson’s disease, these findings suggest that microbial imbalance may be linked to mucosal dysfunction and neuro-immune alterations, providing complementary mechanistic support for the potential benefits of FMT (64).

The stability of the donor microbiota and its persistent colonization in the recipient’s gut are key factors, while individual differences, such as diet, can also affect the sustainability of FMT’s efficacy (54). Therefore, future research should further optimize areas such as donor selection (e.g., microbiota that produce secondary bile acids or butyrate-producing bacteria) (12, 65), transplantation methods, and frequency (66, 67). Additionally, exploring the combined use of dietary interventions, probiotics, or prebiotics could help enhance and maintain the therapeutic effects of FMT (68, 69).

Future research directions include: ① Conducting large-scale, multi-center, randomized controlled trials to authenticate the long-term therapeutic safety and efficacy of FMT. ② Applying multi-omics approaches to elucidate causal relationships within the microbiota–bile acid–receptor axis. ③ Exploring personalized intervention strategies, including efficacy prediction and patient stratification based on bile acid profiles, downstream FXR/TGR5 signaling, and microbiota biomarkers. ④ Combining synthetic biology and customized microbial interventions to advance precision treatment for functional constipation.

Overall, FMT can improve functional constipation by reshaping the gut microbiota, restoring the balance between primary and secondary bile acids, and activating the FXR/TGR5 signaling pathway. Whether as a standalone intervention or in combination with targeted drugs, FMT shows great promise and offers new clinical value for bile acid receptors as therapeutic targets.
